# Urinary 1-aminopyrene level in Koreans as a biomarker for the amount of exposure to atmospheric 1-nitropyrene

**DOI:** 10.1007/s43188-021-00096-z

**Published:** 2021-04-03

**Authors:** Bolormaa Ochirpurev, Sang-Yong Eom, Akira Toriba, Yong-Dae Kim, Heon Kim

**Affiliations:** 1 Department of Preventive Medicine, College of Medicine, Chungbuk National University, 1 Chungdae-ro, Seowon-gu, Cheongju, 28644 Republic of Korea; 2 Chungbuk National University Hospital, 776, 1Sunhwan-ro, Seowon-gu, Cheongju, Republic of Korea; 3 Department of Hygienic Chemistry, Graduate School of Biomedical Science, Nagasaki University, Nagasaki, 852-8521 Japan

**Keywords:** 1-Nitropyrene, Urinary 1-aminopyrene, Diesel exhaust particle, Nitro-PAH

## Abstract

1-Nitropyrene (1-NP) is a major nitro-polycyclic aromatic hydrocarbon (nitro-PAH), and a common constituent in diesel exhaust particles (DEPs). Absorbed 1-nitropyrene is partly metabolized to 1-aminopyrene and excreted in urine. Recently, the number of diesel cars has been increasing, which could be a major cause of air pollution, resulting elevated levels of traffic-related DEPs around cities. The aim of this study was to investigate the usability of 1-aminopyrene (1-AP) as a biomarker for DEP exposure by examining the association between urinary 1-AP concentration and the amount of exposure to atmospheric 1-NP. The study subjects included 65 individuals who work on vehicular roads or bus terminals. Their 24 h urine samples were collected, and atmospheric air was sampled using a personal air sampler for 24 h. Urinary 1-AP and atmospheric nitro-PAH levels were measured using a high-pressure liquid chromatography-fluorescence detector (HPLC-FD). The average urine 1-AP concentration was 0.334 pg/g creatinine. Urinary 1-AP levels were significantly correlated with 1-NP level exposure (r = 0.385, *p* = 0.002) but not with the other nitro-PAHs. When the subjects were classified into high-and low-exposure groups, a significant association was only found in the high exposure group (r = 0.357, *p* = 0.045). In conclusion, there was a significant correlation between 1-NP exposure and urinary 1-AP concentration; therefore, urinary 1-AP level could be used as an exposure biomarker for DEP.

**Supplementary Information:**

The online version contains supplementary material available at 10.1007/s43188-021-00096-z.

## Introduction

1-Nitropyrene (1-NP), an important nitro-polycyclic aromatic hydrocarbon (nitro-PAH) and a major constituent in diesel exhaust particles (DEPs), is mutagenic and carcinogenic to humans and has been classified into group 2A by the International Agency for Research on Cancer [1–4]. Absorbed 1-nitropyrene is partly metabolized to 1-aminopyrene and excreted in urine. Recently, the number of diesel cars has been increasing, which could be a major cause of traffic-related DEP air pollution around cities. Inhaled 1-NP is metabolized by nitro-reduction and P450-mediated ring oxidation and excreted through urine in the form of glucuronide and sulfate. Nine metabolites of 1-NP were detected in in vivo studies: hydroxy-1-nitropyrenes (3-, 6-, and 8-OHNP), hydroxyl-*N*-acetyl-1-aminopyrenes (3-, 6-, and 8-OHNAAP), *N*-acetyl-1-aminopyrene (NAAP), 1-aminopyrene (1-AP), and trans-4,5-dihydro-4,5-dihydroxy-1-nitropyrene) [5–7].

Urinary concentrations of these metabolites can be used to assess exposure to DEP. However, whether the urinary concentrations of these substances are statistically related to DEP exposure levels has not been adequately verified. Although a significant association between urinary 1-AP level and elemental carbon exposure has been reported [8–13], no study has studied the relationship between 1-NP exposure and 1-AP excretion.

The main goal of this study was to investigate the possibility of using 1-AP as a biomarker for DEP exposure by examining the association between 1-AP concentration in urine and the 1-NP exposure level.

## Materials and methods

### Study subjects

This study was conducted after review and approval by the Chungbuk National University Bioethics Committee (IRB). The study subjects were 65 Cheongju residents who were recruited from a group expected to be occupationally exposed to DEP. Among them, 16 were Yakurt delivery and sales females, 8 were bus terminal workers, 37 were street sweepers, and 4 were office workers. They signed the consent form after being informed of the study in detail and provided blood and 24 h urine samples.

A direct interview was conducted using a questionnaire that included questions on demographic characteristics, working environments, dietary patterns, and diesel car use.

The subjects were divided into two groups. Those with 1-NP exposure above average were classified into the high exposure group, and those with below average exposure were classified into the low exposure group.

### Measurement of nitro-PAH concentrations in the atmosphere

Air was sampled at a flow rate of 3 L/min for 24 h with a personal air sampler (Apex standard, SN0376420 Casella CEL, Bedford, England) with a filter holder attached to the collars of the subjects. PTFE filters with a pore size of 2 µm were used. The filters were placed in a flask and mixed with dichloromethane (2 mL). The flask was shaken and treated with ultrasonic waves to extract PAHs and nitro-PAHs. The extracts were evaporated to dryness and the residue was redissolved in acetonitrile. Aliquots of the solution were then injected into a two-dimensional high-performance liquid chromatography (HPLC) system with a fluorescence detector (FD) for the quantification of PAHs and nitro-PAHs [14]. The injected sample was eluted through a clean-up column (Cosmosil, 5NPE, 150 × 4.6 mm i.d., 5 μm, Nacalai Tesque, Kyoto, Japan) with guard column 1 (10 × 4.6 mm i.d.), and nitro-PAHs were reduced to their amino-derivatives using a reduction column (NPpak-RS, 10 × 4.6 mm i.d., JASCO, Tokyo, Japan) at 80 °C. The mobile phase for the clean-up and reduction columns was an ethanol/acetate buffer (pH 5.5) (95/5, v/v) at a flow rate of 0.2 mL/min. A fraction of the amino derivatives and unchanged PAHs eluted from the reduction column with the mobile phase were mixed with 30 mM ascorbic acid at a flow rate of 1.6 mL/min and then trapped on the concentration column (Spheri-5 RP-18, 30 × 4.6 mm i.d. 5 μm, Perkin Elmer, MA, USA). The concentrated fraction was passed through two separation columns (Inertsil ODS-P, 250 × 4.6 mm i.d., 5 μm, GL Sciences, Tokyo, Japan) with a guard column (10 × 4.6 mm i.d.) in tandem. All columns, except the reduction column, were maintained at 20 °C. A programmed gradient elution of the separation columns was performed using 10 mM imidazole buffer (pH 7.6) as eluent A and acetonitrile as eluent B. Finally, the separated analytes were detected with their optimum excitation and emission wavelengths by the dual-channel FD. Eighteen nitro-PAHs, including 1,3-, 1,6-, and 1,8-dinitropyrene (1,3-, 1,6- and 1,8-DNP), 2-nitrofluorene (2-NF), 9-nitrophenathrene (9-NPh), 2- and 9-nitroanthracene (2- and 9-NA), 1-, 2-, and 3-nitrofluoranthrene (1-, 2- and 3-NFR), 1-, 2-, and 4-nitropyrene (1-, 2-, and 4-NP), 7-nitrobenz[*a*]anthracene (7-NBaA), 6-nitrochrysene (6-NC), 6-nitrobenz[*a*]pyrene (6-NBaP), 1-nitroperylene (1-NPer), and 3-nitroperylene (3-NPer), were measured as analytes (Fig. S1).

### Measurement of urinary 1-aminopyrene

**Fig. 1:**
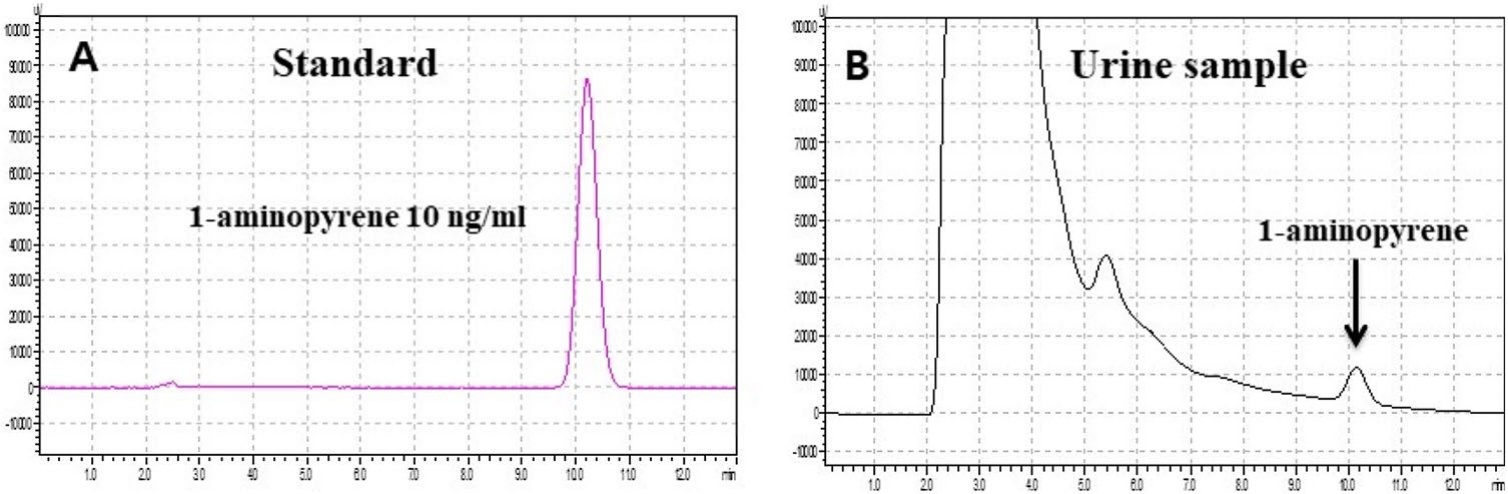
Chromatograms for 1-aminopyrene (**a** 1-aminopyrene standard 10 ng/ml, **b** urine sample)

Urinary 1-AP levels were measured from 2 h urine samples. One milliliter of 10 M hydrochloric acid was added to each 10 mL urine sample, and the urine was stirred in a 90-°C water bath for 2 h. After adjusting the pH to 7.0–8.0, the supernatant was extracted with Sep-Pak cartridge (C18, 3 mL, 200 mg, Waters, Milford, UK). Before extraction, it was pre-conditioned with 5 mL of methanol and 5 mL of water. After loading the sample, it was washed with 5 mL of 20% methanol in water and then extracted with 4 mL of 100% methanol. The extract was dried with N_2_ gas and dissolved in 200 μL of methanol. The extract was analyzed by injecting 20 μL into the HPLC system equipped with a fluorescence detector (Shimadzu, RF-20A, Kyoto, Japan). A reverse phase-amide column (Ascentis RP-Amide, 25 cm × 4.6 mm, 5 μm, SUPELCO, Bellefonte, PA, USA) was used. The mobile phase was composed of methanol and 50 mM sodium acetate buffer pH 7.2 (80:20, v/v) at a flow rate of 1.0 mL/min, and the excitation and emission wavelengths were 254 nm and 425 nm, respectively (Fig. 1). Urinary 1-AP concentration was corrected using the urinary creatinine level.

### Statistical analysis

Statistical analysis was performed using IBM SPSS Statistics 24.0 (IBM Corp., Armonk, NY, USA). The concentrations of nitro-PAH in the air and 1-AP in urine were analyzed for outliers by plotting a scatterplot. To prevent statistical significance distortion by extreme values, the confirmed outliers were replaced with mean + 2 × standard deviation values. The difference between the two groups in the concentration of nitro-PAH in air and the concentration of nitro-PAH metabolites in urine was compared using the Student’s *t* test or Mann–Whitney test, and the comparison between the three groups was analyzed using one-way ANOVA or the Kruskal–Wallis test. The relationship between urinary 1-AP concentration and the exposure levels of nitro-PAHs, including 1-NP, was statistically tested using Pearson’s correlation analysis and a general linear model. All statistical analyses were determined to be statistically significant at a significance level of < 0.05.

## Results

**Fig. 2:**
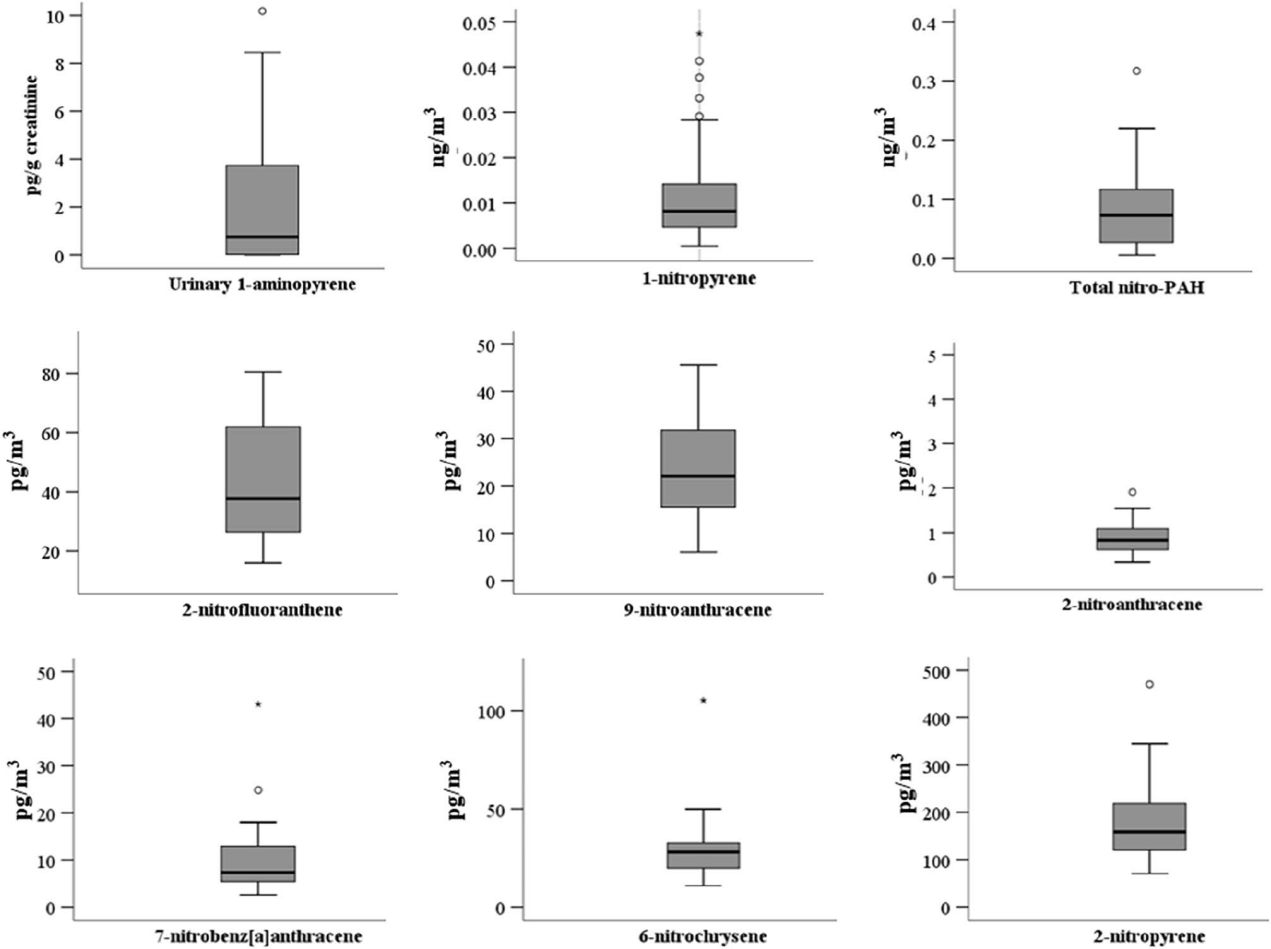
Distribution of urinary 1-aminopyrene and atmospheric nitro-PAH concentrations

Figure 2 shows the distribution of urinary 1-AP and seven atmospheric nitro-PAHs.

The urinary concentration of 1-AP and levels of exposure to 1-NP and total nitro-PAHs according to the demographic characteristics of the study subjects are presented in Table 1. The average 1-AP concentration was 0.334 pg/g creatinine (men: 0.41 pg/g creatinine; women: 0.195 pg/g creatinine). The 1-AP and 1-NP levels did not significantly differ according to gender, smoking habit, occupation, location of residence, driving of diesel cars, participation in outdoor activities, or consumption grilled meat.

**Table 1:** Atmospheric nitro-PAH and urinary 1-AP levels according to general characteristics

Characteristics	No (%)	Geometric mean (95% Confidence interval)					
		1-AP (pg/g creatinine)	*p*	1-Nitropyrene (ng/m^3^)	*p*	Total nitro-PAHs (ng/m^3^)	*p*
All subject	65 (100)	0.334 (0.161, 0.691)		0.009 (0.006, 0.011)		0.053 (0.040, 0.069)	
Occupation			0.232^a^		0.174^a^		0.060^a^
Bus terminal worker	8 (12)	0.413 (0.063, 2.726)		0.005 (0.004, 0.008)		0.108 (0.074, 0.159)	
Street cleaner	37 (57)	0.289 (0.002, 50.717)		0.010 (0.007, 0.014)		0.053 (0.038, 0.073)	
Yakurt delivery ladies	16 (25)	0.425 (0.149, 1.214)		0.006 (0.003, 0.012)		0.036 (0.017, 0.074)	
Office workers	4 (6)	0.179 (0.040, 0.794)		0.018 (0.001, 0.227)		0.059 (0.015, 0.233)	
Sex			0.121^b^		0.968^b^		0.282^b^
Males	47 (72)	0.410 (0.170, 0.992)		0.009 (0.007, 0.013)		0.061 (0.046, 0.080)	
Females	18 (28)	0.195 (0.050, 0.762)		0.007 (0.004, 0.012)		0.036 (0.019, 0.069)	
Smoking status			0.188^b^		0.595^b^		0.102^b^
Non-smokers	34 (52)	0.289 (0.110, 0.757)		0.008 (0.006, 0.011)		0.044 (0.030, 0.065)	
Current or ex-smokers	31 (48)	0.392 (0.123, 1.244)		0.009 (0.006, 0.014)		0.064 (0.044, 0.093)	
Current smoking			0.133^b^		0.092^b^		0.797^b^
No	14 (22)	0.816 (0.107, 6.192)		0.015 (0.007, 0.032)		0.059 (0.031, 0.113)	
Yes	17 (26)	0.214 (0.051, 0.895)		0.006 (0.004, 0.01)		0.069 (0.042, 0.112)	
Road adjacency			0.520^b^		0.564^b^		0.798^b^
< 50 m	39 (60)	0.215 (0.079, 0.582)		0.008 (0.006, 0.011)		0.055 (0.039, 0.077)	
≥ 50 m	26 (40)	0.647 (0.224, 1.870)		0.009 (0.006, 0.015)		0.050 (0.032, 0.078)	
Diesel car ownness			0.127^b^		0.464^b^		0.830^b^
Yes	28 (43)	0.405 (0.119, 1.382)		0.010 (0.006, 0.016)		0.054 (0.036, 0.081)	
No	37 (57)	0.288 (0.114, 0.730)		0.008 (0.005, 0.011)		0.052 (0.036, 0.075)	
Outdoor activity			0.126^b^		0.827^b^		0.876^b^
≤ 8 h	20 (31)	0.306 (0.085, 1.106)		0.007 (0.004, 0.012)		0.053 (0.031, 0.090)	
> 8 h	45 (69)	0.347 (0.139, 0.866)		0.009 (0.007, 0.013)		0.053 (0.038, 0.072)	
Roasted meat consumption			0.140^b^		0.372^b^		0.834^b^
≤ 3 day	37 (57)	0.364 (0.139, 0.952)		0.010 (0.007, 0.014)		0.056 (0.041, 0.078)	
> 3 day	28 (43)	0.297 (0.091, 0.972)		0.007 (0.005, 0.011)		0.048 (0.030, 0.076)	
Season			0.080^b^		0.652^b^		0.256^b^
High	37 (57)	0.186 (0.077, 0.448)		0.008 (0.006, 0.012)		0.060 (0.042, 0.086)	
Low	28 (43)	0.725 (0.213, 2.471)		0.009 (0.006, 0.014)		0.044 (0.030, 0.066)	

ANOVA (a) and *t* test (b) were used for the statistical analysis

The geometric mean of 1-NP and total nitro-PAH exposure levels were 0.009 ng/m^3^ and 0.053 ng/m^3^, respectively. The mean values for males were slightly higher than those for females, but there was no significant difference in the other variables.

The association between quantified nitro-PAHs (9-NA, 2-NA, 2-NFR, 1-NP, 7-NBaA, 6-NC, 2-NP, and total NP) and urinary 1-AP was analyzed using Pearson’s correlation analysis (Table 2). These nitro-PAHs were quantified for all samples, but the others were not detected or were under quantification limits. For all subjects, the correlation coefficients between 1-NP exposure and urinary 1-AP excretion in 2 h urine samples were significant (r = 0.385, *p* = 0.002), especially in the high exposure group. However, 1-AP excretion in the 2 h urine samples did not show a significant correlation with the other nitro-PAHs (Table 2).

**Table 2:** Pearson correlation coefficients between urinary excretion of 1-AP and environmental exposure to nitro-PAHs

Nitro-PAHs	Total			1-NP low exposure			1-NP high exposure		
	n	r	p	n	r	p	n	r	p
9-NA	33	− 0.179	0.320	15	− 0.041	0.885	18	− 0.277	0.267
2-NA	26	− 0.138	0.503	9	0.007	0.986	17	− 0.173	0.506
2-NFR	65	− 0.093	0.461	33	− 0.313	0.076	32	− 0.153	0.403
1-NP	65	0.385	0.002	33	− 0.176	0.328	32	0.357	0.045
7-NBaA	39	− 0.218	0.183	17	− 0.412	0.100	22	− 0.137	0.545
6-NC	40	− 0.184	0.256	19	− 0.509	0.026	21	− 0.138	0.552
2-NP	52	− 0.239	0.088	25	− 0.506	0.010	27	− 0.310	0.116
Total NP	65	0.050	0.690	33	− 0.390	0.025	32	0.009	0.961

A positive association was observed between creatinine-adjusted 1-AP levels and 1-NP exposure levels, in a multivariate analysis that controlled for age, sex, smoking status, roasted meat consumption, occupation, outdoor activity, season, road adjacency, and diesel car ownership (Table 3). As the 1-NP concentration increased by 1 pg/m^3^, the 1-AP concentration increased by 0.262 pg/g creatinine. All other covariates were not significantly correlated with the creatinine-adjusted 1-AP levels (Table 3).

**Table 3:** Parameter estimates for a general linear model for 1-AP (pg/g creatinine)

	beta	p
Intercept	− 0.010	0.785
Age (year)	0.000	0.198
Sex	0.021	0.070
Smoking status (yes, no)	0.010	0.172
Roasted meat consumption (> 3 day)	0.007	0.216
Occupation	− 0.008	0.158
Outdoor activity (> 8 h)	0.003	0.599
Season (high)	− 0.012	0.069
Road adjacency (< 50 m)	0.005	0.354
Diesel car owned (yes)	− 0.005	0.364
1-NP pg/m^3^	0.262	0.001
R^2^	0.312	

## Discussion

This study is the first to simultaneously measure urinary 1-AP excretion and exposure to atmospheric nitro-PAHs in Korean residents. The average 1-NP level was 8.5 pg/m^3^ for all participants in this study. Recently, Hayakawa et al. [15] reported the levels of atmospheric 1-NP and 6-NBaP in five cities, including Busan, South Korea. The median atmospheric 1-NP concentration ranged from 12 to 91 fmol/m^3^, which corresponded to 3.0–22.5 pg/m^3^, and the average 1-NP concentration in the present study was within this range. The median and interquartile range 1-NP levels of US-Mexico border commuters were 0.96 and 0.33–1.87 pg/m^3^, respectively, and those of non-border commuters were 0.15 and 0.05–0.30 pg/m^3^, respectively [16], which were much lower than the mean values of this present study. A 1994 study in Kanazawa (Japan) found that the level of atmospheric 1-NP was 32 pg/m^3^, substantially higher than that in this study, and decreased over time to below the Korean average [17]. However, 1-NP levels in cities in Peru and China were much higher than those in this study. The level of 1-NP was 104 ± 31 pg/m^3^ in Trujillo [12], and approximately 80 pg/m^3^ in Shenyang, China [18].

1-AP levels did not show any differences according to age, sex, smoking status, roasted meat consumption, occupation, outdoor activity, season, road adjacency, and diesel car ownership. Some previous studies have also reported that the level of 1-AP does not statistically differ with smoking status [19, 20].

Neophytou et al. [20] reported that median urinary 1-AP concentrations of 82 male U.S trucking industry workers was 28.7 pg/mg creatinine. The median time-weighted-average concentrations of urinary 1-AP was 138.7 and 21.7 ng g^−1^ creatinine for DEP-exposed subjects and clean air exposed controls [9]. These values were both significantly higher than the mean of the present study.

Assuming that the daily excretion of creatinine in urine is approximately 2 g, the urinary excretion of 1-AP was 8.2 and 3.9 pg in the male and female subjects of this study, respectively. Since the respiratory rate per minute is approximately 7 L, the amount of 1-NP exposed is 90.7 pg and 70.6 pg in males and females, respectively. Therefore, 10.3% and 6.3% of 1-NP in males and females, respectively, was metabolized to 1-AP and excreted via urine. In contrast, the amount of urinary 1-AP excretion was greater than that of 1-NP exposure in most previous studies [9].

We observed a significant relationship between 1-NP exposure and urinary 1-AP excretion in 2 h urine samples. However, 1-AP excretion in the 2 h urine samples did not show a significant correlation with the other nitro-PAHs (9-NA, 2-NA, 2-NFR, 7-NBaA, 6-NC, 2-NP). Interestingly, 1-AP was associated with 1-NP in the high exposure group, but not in the low exposure group (Table 2). A similar significant relationship in a high DEP exposure group has also been reported in a previous study [9].

We observed a positive association between creatinine-adjusted 1-AP and 1-NP exposure levels in a multivariate analysis controlling for age, sex, smoking status, road adjacency, season, and diesel car ownership. All other covariates were not significantly correlated with the creatinine-adjusted 1-AP levels (Table 3). Studies on traffic workers [8, 10, 16] have shown a positive correlation between 1-NP exposure levels and urinary concentrations of 1-NP metabolites (6-OHNP, 8-OHNP, and 8-OHNAAP); however, 1-AP was not measured in these studies.

The limitations of this study were that there was an insufficient number of subjects, and the distinction between the exposed and control groups was not clear.

This cross-sectional design was another limitation of this study. The peak urinary 1-AP excretion time could be longer than 24 h after exposure. To determine when the urinary excretion of 1-AP is highest after exposure to 1-NP, urinary 1-AP levels in serial urine samples should be measured. In this study, however, only urinary excretion of 1-AP between 22 and 24 h after exposure was measured. Furthermore, there could be individual differences in the metabolic rate of 1-NP; however, this polymorphism was not controlled for in our study.

## Supplementary Information

Below is the link to the electronic supplementary material.

Supplementary file1 (DOCX 105 KB)
