# Supplementary material for: Urinary 1-aminopyrene level in Koreans as a biomarker for the amount of exposure to atmospheric 1-nitropyrene
Source: Toxicol Res. 2021 Apr 3;38(1):45–51. doi: 10.1007/s43188-021-00096-z (PMC8748593; doi:10.1007/s43188-021-00096-z)
Supplement: Supplementary file 1 — Supplementary file1 (DOCX 105 KB) [file 43188_2021_96_MOESM1_ESM.docx]

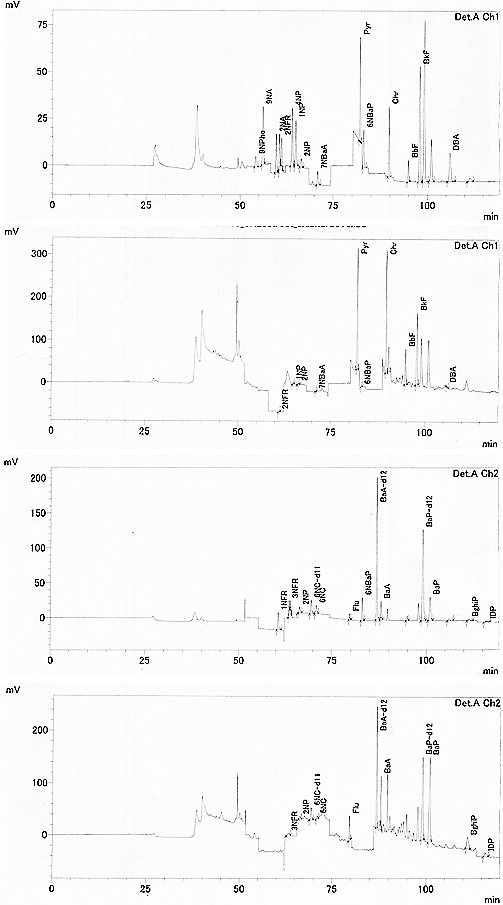


Figure S1. Representative two-channel HPLC chromatograms of a standard for PAHs and nitro-PAHs and an extract of atmospheric sample collected by the personal air sampler.
